# Clinical and Economic Outcomes in Patients With Metastatic Urothelial Carcinoma Receiving First-Line Systemic Treatment (the IMPACT UC I Study)

**DOI:** 10.1093/oncolo/oyad174

**Published:** 2023-07-11

**Authors:** Mehmet A Bilen, Scott B Robinson, Amy Schroeder, Jing Peng, Ruth Kim, Frank X Liu, Abhijeet Bhanegaonkar

**Affiliations:** Winship Cancer Institute of Emory University, Atlanta, GA, USA; Department of Hematology and Medical Oncology, Emory University School of Medicine, Atlanta, GA, USA; Inovalon, Bowie, MD, USA; Avalere Health, Washington, DC, USA; Avalere Health, Washington, DC, USA; Pfizer, New York, NY, USA; EMD Serono, Inc., Rockland, MA, USAan affiliate of Merck KGaA; EMD Serono, Inc., Rockland, MA, USAan affiliate of Merck KGaA

**Keywords:** metastatic urothelial carcinoma, Medicare, first-line treatment, immune checkpoint inhibitors, treatment patterns, healthcare resource utilization

## Abstract

**Background:**

The IMPACT UC I study assessed real-world treatment patterns, outcomes, healthcare resource utilization (HCRU), and costs in patients with metastatic urothelial carcinoma (mUC) receiving first-line (1L) systemic treatment after the FDA approval of 1L immune checkpoint inhibitor (ICI) monotherapy.

**Patients and Methods:**

This retrospective study used 100% Medicare fee-for-service claims from 1/1/2015 to 6/30/2019 to identify patients aged ≥18 years diagnosed with UC with evidence of metastatic disease, continuously enrolled for 6 months before and after initial diagnosis. Patients were grouped by 1L treatment: cisplatin-containing chemotherapy, carboplatin-containing chemotherapy, ICI monotherapy, or nonplatinum-containing therapy. Unadjusted time on 1L treatment (TOT), overall survival (OS), HCRU, and total healthcare costs were analyzed.

**Results:**

Of 18 888 patients with mUC, 8630 (45.7%) had received identified 1L systemic treatment; platinum-containing chemotherapy was the most common (cisplatin-containing chemotherapy, 37.6%; carboplatin-containing chemotherapy, 30.2%). Cisplatin- and carboplatin-containing chemotherapy had the shortest time-to-treatment initiation (median, 1.7-3.0 months) and longest TOT (median, 4.0-4.3 months). Median OS was longest with cisplatin-containing chemotherapy (20.0 months) and shortest with ICI monotherapy (7.6 months). Cisplatin- and carboplatin-containing chemotherapy were associated with highest HCRU; total healthcare costs were approximately 2-fold higher with ICI monotherapy vs other 1L treatments ($10 359 vs $5042-$5709 per patient per month).

**Conclusion:**

1L platinum-containing chemotherapy resulted in the longest median OS and highest HCRU, whereas 1L ICI treatment had the shortest median OS and the highest costs. Over 50% of patients diagnosed with advanced UC (aUC) received no systemic therapy, highlighting the importance of optimal 1L treatment decisions in aUC.

## Teaser

The IMPACT UC I study assessed real-world treatment patterns, outcomes, healthcare resource utilization, and costs in patients with metastatic urothelial carcinoma receiving first-line systemic treatment after the FDA approval of first-line immune checkpoint inhibitor monotherapy.

**Implications for Practice**

There are different treatments available for people with bladder cancer that has spread to other parts of the body, known as metastatic bladder cancer. The first treatment that people receive is called first-line treatment. This study looked at Medicare fee-for-service claims between 2015 and 2019 to identify people with metastatic bladder cancer to see patterns and outcomes in the first-line treatments used, in addition to the healthcare resources and costs associated with them. Results from this study highlight the importance of careful and considerate decision-making for first-line treatments for people with metastatic bladder cancer.

## Introduction

Bladder cancer is the sixth most commonly occurring cancer in the US, with approximately 81 000 new cases and 17 000 deaths estimated to occur in 2022.^1^ Factors associated with an increased risk of developing bladder cancer include male sex, smoking, and age ≥55 years; median age at diagnosis is 72 years.^2,3^ In the US, bladder cancer is approximately twice as likely to occur in White men compared with African American or Hispanic men.^3^ Approximately 5% of patients have metastatic disease at diagnosis, for which prognosis is poor; including 5-year survival rates of <7.7%.^1^ Urothelial carcinoma (UC), which originates in the cells that line the urinary tract (including the bladder, ureters, renal pelvis, and urethra), is the most common form of bladder cancer, accounting for >90% of cases.^4^

UC is considered a chemotherapy-sensitive tumor, and platinum-containing chemotherapy continues to be the standard-of-care treatment in the first-line (1L) setting.^4-6^ US National Comprehensive Cancer Network (NCCN) Guidelines recommend 1L treatment with either cisplatin- or carboplatin-containing chemotherapy depending on patient cisplatin eligibility.^4^ For cisplatin-eligible patients, recommended 1L treatments (category 1) are either ≤6 cycles of cisplatin plus gemcitabine, or a dose-dense combination of methotrexate, vinblastine, doxorubicin and cisplatin.^4-6^ For patients who are unable to receive cisplatin because of poor performance status, renal impairment, hearing loss, peripheral neuropathy, symptomatic heart failure, or other comorbidities, recommended chemotherapy is carboplatin plus gemcitabine.^4,7^

UC is an immunogenic tumor, providing the basis for the activity of immune checkpoint inhibitors (ICI) in the treatment of patients with advanced UC.^4,8-12^ Atezolizumab and pembrolizumab received accelerated US Food and Drug Administration (FDA) approval in 2017 for 1L treatment of cisplatin-ineligible patients with advanced UC, which was restricted in 2018 to cisplatin-ineligible patients with programmed cell death 1 ligand 1 (PD-L1)–positive tumors or platinum-ineligible patients.^4,13-15^ In 2021, the accelerated approval of pembrolizumab was converted to a full approval, but its 1L indication was further restricted to platinum-ineligible patients only.^14,16^ In 2020, avelumab, an anti–PD-L1 ICI, was approved by the FDA as 1L maintenance treatment for patients with advanced UC that had not progressed with 1L platinum-containing chemotherapy.^4^ This approval was based on outcomes in the phase 3 JAVELIN Bladder 100 trial (NCT02603432), in which avelumab 1L maintenance plus best supportive care significantly prolonged overall survival (OS) and progression-free survival vs best supportive care alone.^17^ These results supported the inclusion of avelumab 1L maintenance in the NCCN Guidelines as a category 1 recommendation for cisplatin-eligible or -ineligible patients with advanced UC that has not progressed with 1L platinum-containing chemotherapy, irrespective of PD-L1 status.^4^

Limited real-world data exist documenting changes in clinical and economic outcomes following the implementation of 1L ICI monotherapy into clinical practice for metastatic UC. IMPACT UC I is a retrospective study evaluating real-world treatment patterns, clinical outcomes, HCRU, and costs associated with 1L systemic treatment for patients with metastatic UC during the period after the introduction of 1L ICIs but before the approval of avelumab 1L maintenance.

## Patients and Methods

### Study Design and Data Source

IMPACT UC I was a retrospective cohort study utilizing de-identified 100% Medicare fee-for-service (FFS) claims data to identify patients with metastatic UC who received 1L treatment. Access to Medicare FFS claims was permitted through a research-focused data use agreement with the Centers for Medicare & Medicaid Services (CMS). Claims data included information about patient demographics, diagnosis and procedure codes, and dates of admission, discharge, death, and service for visits. Inpatient and outpatient data were derived from Parts A and B and prescription drug event data from Part D of the claims. This work did not require institutional review board approval.

### Patient Population

**Figure 1.**
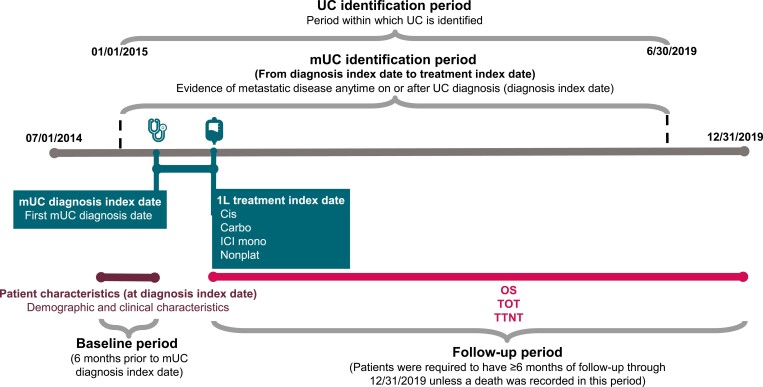
Study design. Abbreviations: 1L, first line; carbo, carboplatin-containing chemotherapy; cis, cisplatin-containing chemotherapy; ICI, immune checkpoint inhibitor; mUC, metastatic urothelial carcinoma; nonplat, nonplatinum-containing chemotherapy; OS, overall survival; TOT, time on treatment; TTNT, time to next treatment; UC, urothelial carcinoma.

Patients included in the study population were aged ≥18 years with a diagnosis code for UC in any position within the claim based on International Classification of Disease (ICD) coding conventions (prior to October 1, 2015: *ICD, 9th Revision (ICD-9),* 188.x-189.x, except 189.0; after September 30, 2015: *ICD, 10th Revision (ICD-10):* C65.x-C68.x; note that x indicates a wildcard designating any subcode). All patients were required to have ≥1 inpatient or emergency department visit or ≥2 outpatient medical visits ≥7 days apart between January 1, 2015, and June 30, 2019. The index diagnosis date was defined as the date of the first diagnosis of UC. Patients were required to have ≥6 months continuous enrollment for medical and pharmacy benefits prior to the index diagnosis date to identify baseline characteristics (baseline period). Patients were also required to have a diagnosis code for secondary malignant neoplasm (prior to October 1, 2015: *ICD-9*, 196.x-198.x; after September 30, 2015: *ICD-10* C77.x-C79.x.) on or any time after the index diagnosis date to identify metastatic disease. Patients were assumed to have received treatment for metastatic disease if they had diagnosis codes for both UC and metastatic disease. Patients with evidence of prior primary cancers were excluded. The index treatment date was defined as the start date of 1L treatment (ie, date of the first claim for 1L treatment for metastatic UC following the index diagnosis date for metastatic UC). The post–index treatment follow-up period was measured from the index treatment date through the end of the study (December 31, 2019), death, or disenrollment to capture information related to HCRU with a follow-up of ≥6 months. Figure 1 summarizes the study timeline.

### Treatment Categories and Data Collection

Patients with metastatic UC with evidence of 1L treatment were categorized into 1 of 4 mutually exclusive cohorts based on type of 1L treatment received: cisplatin-containing chemotherapy, carboplatin-containing chemotherapy, ICI monotherapy, or nonplatinum-containing therapy (excluding ICIs). Information regarding baseline demographics was collected during the baseline period. Information for each line of treatment was collected within 30 days of the first drug claim for that line.

### Line of Treatment Algorithm

Regimens for each line of treatment were determined based on all drugs received within 30 days of the first drug claim. Subsequent lines were defined as new treatments received ≥30 days after the first drug claim. Specified circumstances where change of treatment was not defined as a new line of treatment were as follows: switch from cisplatin-containing to carboplatin-containing chemotherapy (these patients were categorized into the carboplatin-containing chemotherapy cohort); switch from one ICI to another ICI within 30 days; and switch from paclitaxel to albumin-bound paclitaxel within 30 days. Any gap in treatment ≥60 days was considered as termination of the prior line of treatment, even if the same treatment was received in the subsequent line.

### Clinical Outcomes

Analyses included time on 1L treatment (TOT; defined as time between 1L treatment initiation and discontinuation) and treatment-free interval (TFI; defined as time between end of 1L treatment and 2L treatment initiation). OS was measured from date of treatment initiation to date of death, validated by CMS. If no date of death was found, the patient was assumed to be alive or lost to follow-up and was censored at end of enrollment or end of study, whichever occurred first.

### HCRU and Cost Outcomes

HCRU and costs were collected for all-cause encounters across the treatment follow-up period. HCRU was measured per 100 patients per month (P100PPM). All-cause HCRU was defined as the number of inpatient (including median length of stay), emergency department, and outpatient visits. Information regarding the number of patients with claims for skilled nursing facilities, hospices, and home health was also identified, including number of stays. Costs were measured per patient per month (PPPM). Total costs included all costs associated with medical and pharmacy claims across the treatment follow-up period. Total medical costs included all costs associated with inpatient hospitalization, outpatient, and post–acute care claims. Inpatient hospitalization costs included all costs associated with any claims related to inpatient hospitalizations. Similarly, outpatient costs were those associated with any claims related to outpatient visits, including infusions for treatments (such as ICIs and systemic therapies). Post–acute care costs included all costs associated with claims related to home health, skilled nursing, and hospice care. Emergency department costs were a subset of outpatient costs that were identified with a revenue center code for emergency department.

### Statistical Analysis

Descriptive statistics for continuous variables were reported as medians with interquartile ranges. Frequencies and proportions were reported for categorical variables. The Kaplan-Meier method was used to analyze time-to-event data. Statistical significance was defined as *P* < .05. All analyses were performed used SAS Enterprise Guide 7.1 software (SAS Institute; Cary, NC) and SQL Aginity Workbench software (Coginiti; Evanstown, IL).

## Results

### Patients

**Figure 2.**
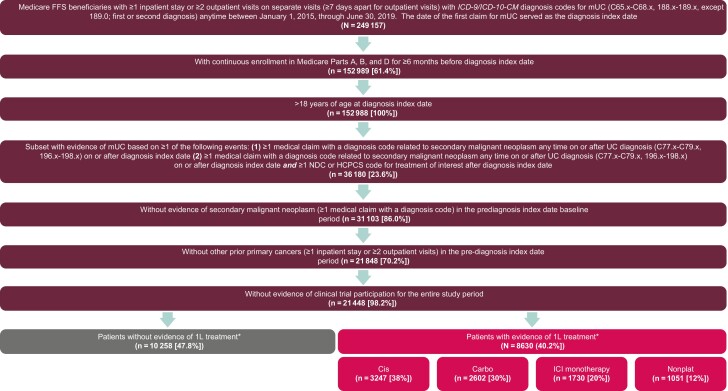
Patient attrition. 1L, first line; 2L, second line; carbo, carboplatin-containing chemotherapy; cis, cisplatin-containing chemotherapy; CM, clinical modification; dID, date of index diagnosis; FFS, fee for service; HCPCS, Healthcare Common Procedure Coding System; *ICD-9*, *International Classification of Diseases, Ninth Revision*; *ICD-10*, *International Classification of Diseases, Tenth Revision*; ICI, immune checkpoint inhibitor; mUC, metastatic urothelial carcinoma; NDC, National Drug Code; nonplat, nonplatinum-containing chemotherapy; UC, urothelial carcinoma. ^*^ Without evidence of prior ICI monotherapy (avelumab, pembrolizumab, nivolumab, atezolizumab, durvalumab) in the pre-dID baseline period. Atezolizumab and durvalumab applications for 2L indication in locally advanced UC and mUC were recently withdrawn.^4^

Of 18 888 patients who met the study criteria, 8630 (45.7%) had received 1L systemic treatment and were included in the study population; 10 258 (54.3%) had no identified 1L systemic treatment. Among those who received 1L treatment, 3247 (37.6%) received cisplatin-containing chemotherapy, 2602 (30.2%) received carboplatin-containing chemotherapy, 1730 (20.0%) received ICI monotherapy, and 1051 (12.2%) received nonplatinum-containing therapy (excluding ICIs; Figure 2; Supplemental Table S1). Of patients who received 1L systemic treatment, median age was 75 years (interquartile range [IQR], 70-80 years), most patients were male (70.4%) and White (90.4%; Table 1). Patient baseline characteristics were mostly consistent across 1L treatment cohorts. Overall, 50.7% had a Charlson Comorbidity Index (CCI) score of >4; patients who received 1L cisplatin-containing chemotherapy had the lowest CCI score (mean, 3.9; standard deviation [SD], 2.6), while those who received 1L ICI monotherapy had the highest CCI score (mean, 5.0; SD, 3.2; Supplementary Figure S1 and Table S2).

**Table 1.** Patient baseline characteristics by 1L treatment cohort.

	1L treatment cohort				
	Cisplatin-containing chemotherapy (*n* = 3247)	Carboplatin-containing chemotherapy (*n* = 2602)	ICI monotherapy (*n* = 1730)	Nonplatinum-containing treatment (*n* = 1051)	No 1L treatment identified (*n* = 10 258)
Median age at index (IQR), years	76 (72-76)	75 (70-81)	79 (73-84)	78 (72-83)	78 (72-85)
Male, *n* (%)	2287 (70.4)	1830 (70.3)	1199 (69.3)	763 (72.6)	6887 (67.1)
Race, *n* (%)					
White	2951 (90.9)	2334 (89.7)	1556 (89.9)	959 (91.2)	9128 (89.0)
Black	139 (4.3)	136 (5.2)	80 (4.6)	46 (4.4)	638 (6.2)
Hispanic or Latino	37 (1.1)	25 (1.0)	NR	NR	129 (1.3)
Other	38 (1.2)	45 (1.7)	41 (2.4)	18 (1.7)	175 (1.7)
Unknown	82 (2.5)	62 (2.4)	39 (2.3)	21 (2.0)	188 (1.8)
US geographic region, *n* (%)					
Midwest	826 (25.4)	662 (25.4)	410 (23.7)	272 (25.9)	2586 (25.2)
Northeast	750 (23.1)	595 (22.9)	403 (23.3)	279 (26.5)	2594 (25.3)
South	1119 (34.5)	959 (36.9)	596 (34.5)	339 (32.3)	3419 (33.3)
West	547 (16.8)	383 (14.7)	320 (18.5)	160 (15.2)	1648 (16.1)
Dual eligible (Medicaid) status, *n* (%)	538 (16.6)	425 (16.3)	282 (16.3)	184 (17.5)	2214 (21.6)
Original reason for entitlement to Medicare, *n* (%)					
Age	2699 (83.1)	2190 (84.2)	1513 (87.5)	892 (84.9)	8476 (82.6)
Disability and/or ESRD	548 (16.9)	412 (15.8)	217 (12.5)	159 (15.1)	1782 (17.4)

Values <11 or that can be calculated to be <11 are suppressed to comply with NCI privacy rules.

Abbreviations: 1L, first line; ESRD, end-stage renal disease; ICI, immune checkpoint inhibitor; IQR, interquartile range; NR, not reported.

### Treatment Patterns

Of patients who received 1L treatment, TOT was longest in those who received carboplatin-containing chemotherapy (4.3 months [IQR, 2.3-9.2 months]) and cisplatin-containing chemotherapy (4.0 months [IQR, 2.6-10.7 months]; Table 2). For patients who received 2L treatment, median TFI between 1L and 2L therapy was 3.7 months for 1L carboplatin-containing chemotherapy (IQR, 2.1-7.3 months) and 3.1 months for 1L cisplatin-containing chemotherapy (IQR, 2.1-4.8 months; Table 2).

**Table 2.** Treatment patterns by 1L treatment cohort.

	1L treatment cohort			
	Cisplatin-containing chemotherapy (*n* = 3247)	Carboplatin-containing chemotherapy (*n* = 2602)	ICI monotherapy (*n* = 1730)	Nonplatinum-containing treatment (*n* = 1051)
Median follow-up (IQR), months	18.6 (10.9-33.1)	17.4 (9.9-30.7)	19.3 (9.7-33.5)	22.1 (11.7-38.0)
Time on 1L treatment (IQR), months	4.0 (2.6-10.7)	4.3 (2.3-9.2)	3.4 (1.6-7.8)	2.5 (1.6-6.7)
Treatment-free interval between 1L and 2L (IQR), months	3.1 (2.1-4.8)	3.7 (2.1-7.3)	3.3 (2.3-9.0)	3.5 (2.3-8.3)

Abbreviations: 1L, first line; 2L, second line; ICI, immune checkpoint inhibitor; IQR, interquartile range.

### Overall Survival

**Figure 3.**
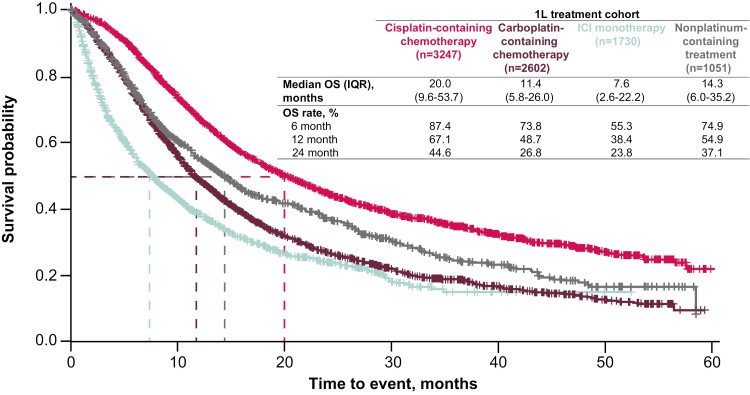
Unadjusted analyses of OS by 1L treatment cohort. 1L, first line; ICI, immune checkpoint inhibitor; IQR, interquartile range; OS, overall survival. OS was measured from initiation of 1L treatment.

In unadjusted analyses, patients who received cisplatin-containing chemotherapy had the longest median OS (20.0 months [IQR, 9.6-53.7 months]), with 6-, 12-, and 24-month OS rates of 87.4%, 67.1%, and 44.6%, respectively (Figure 3). The median OS for patients who received carboplatin-containing chemotherapy was 11.4 months (IQR, 5.8-26.0 months), with 6-, 12-, and 24-month OS rates of 73.8%, 48.7%, and 26.8%, respectively. Patients who received 1L ICI monotherapy had shorter median OS (7.6 months [IQR, 2.6-22.2 months]), with 6-, 12-, and 24-month OS rates of 55.3%, 38.4%, and 23.8%, respectively. Median OS with nonplatinum-containing therapy was 14.3 months (IQR, 6.0-35.2 months).

### HCRU and Costs

Prior to 1L treatment (ie, treatment index date), HCRU was similar between treatment cohorts (data not shown). After the treatment index date (Table 3), the number of outpatient visits for patients receiving cisplatin-containing chemotherapy was 177 P100PPM (IQR, 96-287 P100PPM), for patients receiving 1L carboplatin-containing chemotherapy was 175 P100PPM (IQR, 88-293 P100PPM), and for patients receiving 1L ICI monotherapy was 168 P100PPM (IQR, 86-271 P100PPM). Patients who received 1L carboplatin-containing chemotherapy had the highest number of inpatient visits (median, 15 P100PPM (IQR, 4-32 P100PPM), followed by those who received 1L ICI monotherapy (14 P100PPM [IQR, 0-44 P100PPM]) (Table 3). The median duration of inpatient encounters was similar across all 1L treatment cohorts (4-5 days [IQR, 3-7 days]). Patients who received 1L platinum-containing chemotherapy had the highest median number of emergency department visits (carboplatin-containing chemotherapy, 6 P100PPM [IQR, 0-18 P100PPM]; cisplatin-containing chemotherapy, 6 P100PPM [IQR, 0-16 P100PPM]); the median number of emergency department visits for those who received 1L ICI monotherapy was 0 (IQR, 0-19.4 P100PPM).

**Table 3.** HCRU P100PM post-index date by 1L treatment cohort

	1L treatment cohort			
	Cisplatin-containing chemotherapy (n=3247)	Carboplatin-containing chemotherapy (n=2602)	ICI monotherapy (n=1730)	Nonplatinum- containing treatment (n=1051)
Inpatient hospitalizations				
Patients, *n* (%)	2683 (82.6)	2036 (78.2)	1108 (64)	783 (74.5)
Median number of stays P100PPM (IQR), *n*	13 (4-27)	15 (4-32)	14 (0-44)	12 (0-31)
Median length of stay (IQR), days	5 (3-7)	4 (3-7)	5 (3-7)	4 (3-7)
Emergency department				
Patients, *n* (%)	2005 (61.7)	1538 (59.1)	814 (47.1)	585 (55.7)
Median number of visits P100PPM (IQR), *n*	6 (0-15)	6 (0-18)	0 (0-19)	5 (0-16)
Outpatient				
Patients, *n* (%)	3195 (98.4)	2535 (97.4)	1629 (94.2)	1018 (96.9)
Median number of visits P100PPM (IQR), *n*	177 (96-287)	175 (88-293)	168 (86-271)	171 (89-275)
Skilled nursing facility				
Patients, *n* (%)	686 (21.1)	609 (23.4)	337 (19.5)	243 (23.1)
Median number of stays P100PPM (IQR), *n*	0	0	0	0
Median length of stay (IQR), days	14 (8-25)	15 (8-24)	14 (7-23)	14 (8-26)
Hospice				
Patients, *n* (%)	1318 (40.6)	1353 (52.0)	851 (49.2)	479 (45.6)
Median number of stays P100PPM (IQR), *n*	0 (0-7)	3 (0-11)	0 (0-23)	0 (0-11)
Median length of stay (IQR), days	9 (4-24)	10 (4-30)	11 (4-29)	11 (4-31)
Home health agency				
Patients, *n* (%)	1872 (57.7)	1268 (48.7)	758 (43.8)	514 (48.9)
Median number of stays P100PPM (IQR), *n*	25.5 (0-120)	0 (0-123)	0 (0-164)	0 (0-124)
				

Abbreviations: 1L, first line; HCRU, healthcare resource utilization; ICI, immune checkpoint inhibitor; IQR, interquartile range; P100PM, per 100 patients per month.

In analyses of post–index date costs, in US dollars, patients who received 1L ICI monotherapy had approximately double the total healthcare costs of other 1L treatment cohorts (median, $10 359 PPPM vs $5042–$5709 PPPM) (Table 4). Median medical costs accounted for 92.4%–94.1% of total costs for 1L cisplatin-containing chemotherapy, 1L carboplatin-containing chemotherapy, and nonplatinum-containing treatment, and 97.6% of total costs for 1L ICI monotherapy. Outpatient costs were also substantially higher for 1L ICI monotherapy (median, $4933 PPPM) vs other 1L treatment cohorts ($1290–$1430 PPPM). Median emergency department costs, which were part of outpatient costs, accounted for <1% of total healthcare costs for each 1L treatment cohort, with a median cost of $0 for those receiving 1L ICI monotherapy. Costs for post–acute care, which included skilled nursing facilities, home-health services, and hospices, were highest for 1L ICI monotherapy (median, $584 PPPM) and lowest for 1L carboplatin-containing chemotherapy (median, $256 PPPM). Inpatient hospitalization and pharmacy costs were similar across 1L treatment cohorts and ranged from $1461 to $1842 PPPM and $92 to $130 PPPM, respectively.

**Table 4.** Median costs post–index date (all visits) PPPM.

$ (IQR)	1L treatment cohort			
	Cisplatin-containing chemotherapy (*n* = 3247)	Carboplatin-containing chemotherapy (*n* = 2602)	ICI monotherapy (*n* = 1730)	Nonplatinum-containing treatment (*n* = 1051)
Total healthcare cost	5141 (2480-8799)	5709 (2909-9255)	10 359 (5434-15 115)	5042 (2394-8897)
Total medical cost	4838 (2294-8538)	5365 (2685-8940)	10 115 (5096-14 652)	4662 (2153-8462)
Inpatient hospitalization cost	1842 (555-4118)	1749 (331-4138)	1549 (0-5062)	1461 (0-3858)
Outpatient cost	1397 (552-3174)	1430 (555-3568)	4933 (724-9420)	1290 (554-2881)
Emergency department cost^a^	33 (0-139)	31 (0-158)	0 (0-162)	23 (0-139)
Post–acute care cost^b^	256 (0-843)	408 (0-1252)	584 (0-1837)	373 (0-1292)
Total pharmacy cost	109 (40-257)	112 (39-277)	92 (26-277)	130 (46-294)

^a^Emergency department is part of outpatient costs.

^b^Includes skilled nursing facilities, home health, and hospices.

Abbreviations: 1L, first line; ICI, immune checkpoint inhibitor; IQR, interquartile range; PPPM, per patient per month.

## Discussion

This real-world, retrospective database study of administrative claims data assessed treatment patterns and clinical outcomes, in addition to HCRU and healthcare costs, associated with 1L treatment for metastatic UC from 2015 to 2019 using Medicare 100% FFS claims data in the US. This study provides insights into the treatment landscape of metastatic UC during this period, capturing approvals of 1L ICI monotherapies for cisplatin-ineligible patients in 2017.

Of patients who met study criteria, 54.3% did not receive any 1L systemic treatment, similar to reports from previous real-world studies.^18-20^ The 45.7% of patients who had received 1L systemic treatment were analyzed in detail according to the type of 1L treatment received. Patient baseline characteristics were generally similar between all 1L treatment cohorts and were consistent with expectations for a Medicare FFS population, and most patients had demographic risk factors associated with bladder cancer, ie, older age, male sex, and White ethnicity.^2,3^ As expected, patients who received 1L cisplatin-containing chemotherapy had fewer comorbidities than those who received other 1L treatments; this observation is consistent with specified comorbidities being used to determine ineligibility for cisplatin-containing treatment.^4,7^

In analyses of 1L treatment patterns, patients who re­­­ceived 1L platinum-containing chemotherapy (cisplatin- or carbo­­platin-containing) had the longest TOT compared with other 1L treatment groups. Patients who received 1L platinum-containing chemotherapy had longer median OS than those who received 1L ICI monotherapy in this real-world setting. These results are consistent with those from recent phase III trials as ICI monotherapy did not significantly improve OS vs platinum-containing chemotherapy in the 1L setting.^21-23^ Median OS in this study is likely to have been influenced by patient selection, including the approved indications of 1L ICI monotherapy being limited to either cisplatin-ineligible patients with PD-L1–positive tumors, who have a higher comorbidity burden.^14,15^ However, these results highlight the importance of optimal treatment selection for patients with metastatic UC in the 1L treatment setting and suggest that platinum-containing chemotherapy should be considered for all eligible patients, consistent with the recommendation in international treatment guidelines.^4-6^ This finding is particularly important following the approval of avelumab 1L maintenance, which has been shown to significantly extend OS for patients whose disease had not progressed with 1L platinum-containing chemotherapy.^4,17^

UC is generally associated with a substantial economic burden, particularly in patients with metastatic UC.^24^ In this study, HCRU associated with outpatient emergency department visits was highest in the 1L platinum-containing chemotherapy cohorts due to the longer OS for patients in these cohorts. The proportions of patients who required inpatient hospitalizations in the 1L platinum-containing chemotherapy cohorts (78%–83%) were lower than those reported in a previous study using data from 2004 to 2011 (97%), potentially suggesting improvements in AE management over time with 1L platinum-containing chemotherapy.^20^ In all cohorts, the majority of total healthcare costs was associated with medical costs, which were highest for 1L ICI monotherapy compared with other 1L treatment cohorts; this may be due to the higher number of administrations required for these treatments. Consequently, total healthcare cost for 1L ICI monotherapy was almost double that of other 1L treatments. However, a previous analysis concluded that ICI monotherapy with pembrolizumab was a cost-effective option in cisplatin-ineligible patients with PD-L1–positive tumors.^25^ It should also be noted that the JAVELIN Bladder 100 regimen (1L platinum-containing chemotherapy followed by avelumab 1L maintenance in patients without disease progression) is considered a cost-effective option as patients who have disease control with 1L chemotherapy are more likely to benefit from ICI monotherapy than patients who are treated with ICI monotherapy from diagnosis.

This study has various limitations that should be acknowledged. Firstly, the study population comprises Medicare FFS beneficiaries aged ≥65 years, who may not be reflective of younger patients or patients with other insurance types. This analysis included 100% of Medicare FFS claims for patients diagnosed with bladder cancer between 2015 to 2019; this data was the most recent available at the time of the analysis. Avelumab as first-line maintenance treatment was approved in the US for patients with locally advanced or metastatic urothelial carcinoma without progression following 1L platinum-containing chemotherapy in June 2020,^26^ and has become a standard-of-care regimen.^4-6^ Therefore, future studies should evaluate avelumab first-line maintenance in this population. Furthermore, Medicare insurance is generally only available for people aged 65 years and over, with an average age at diagnosis of 73 years; therefore, these data may not be representative of patients aged less than 65 years. This younger population may have other insurance coverage, and future study of this patient population may reveal different treatment patterns and outcomes. Additionally, because this analysis is based on claims data, the potential exists for miscoding of disease type and misclassification of line of treatments using algorithm-defined criteria. Claims data also provided limited or no information regarding PD-L1 status or cisplatin eligibility. Although data regarding pharmacy prescription fills were obtained, these data included only orally administered treatments and did not confirm whether treatments were taken as prescribed. Additionally, our data do not capture patient preferences for treatment, reasons for assigning a line of therapy or for selection of specific treatments, treatment modifications, treatment tolerability, patient performance status, or other patient characteristics. As such, direct causality cannot be established between treatment category and patient outcomes or HCRU and cost of care. Moreover, this study simply analyzed Medicare FFS claims and did not capture secondary insurance; therefore, the costs reported may be underestimated for some patients. Because of coding practices, rates of health events may also be underestimated. Lastly, causes of death were not captured.

This study provides a benchmark to understand changes in the treatment landscape for metastatic UC following the introduction of 1L ICI monotherapy in clinical practice. Since this study was conducted, the US approval of pembrolizumab has been restricted to platinum-ineligible patients (irrespective of PD-L1 status) and results from several phase III clinical trials of different 1L ICI-containing regimens in patients with advanced UC have been reported.^14,21–23^ Of these, the JAVELIN Bladder 100 trial of avelumab 1L maintenance was the only phase III trial of an ICI to report a significant improvement in OS in the 1L treatment setting, resulting in regulatory approvals and updated treatment guidelines.^4-6,17,27^ Future real-world analyses are warranted to further assess the clinical and health-economic impact of avelumab 1L maintenance as a new standard of care for advanced UC.

## Conclusion

To our knowledge, the IMPACT UC I study is one of the first real-world analyses to use Medicare FFS claims data to assess treatment patterns following the approval of ICIs for the treatment of metastatic UC. This study provides relevant information for healthcare providers, payers, and other stakeholders about patient characteristics, treatment patterns, clinical outcomes, HRCU, and healthcare costs for 1L treatment of metastatic UC in a real-world setting. Notably, approximately half of patients with evidence of metastatic UC did not have any identified 1L systemic treatment, highlighting an unmet medical need in this population. Outcomes from this study also underscore the need for improved education of physicians and patients to optimize treatment selection in the 1L setting for a long-term clinical benefit as treatment guidelines suggest that platinum-containing chemotherapy should be considered for all eligible patients.^4-6^ This may be more important following the outcomes of the JAVELIN Bladder 100 trial; these results have subsequently led to the use of avelumab 1L maintenance after 1L platinum-containing chemotherapy as a standard of care for eligible patients with advanced UC. These data provide a benchmark for future studies to assess the evolving treatment landscape.

## Supplementary Material

oyad174_suppl_Supplementary_Materials

## Data Availability

No new data were generated or analyzed in support of this research.
