# Supplementary material for: Clinical and Economic Outcomes in Patients With Metastatic Urothelial Carcinoma Receiving First-Line Systemic Treatment (the IMPACT UC I Study)
Source: Oncologist. 2023 Jul 11;28(9):790–8. doi: 10.1093/oncolo/oyad174 (PMC10485286; doi:10.1093/oncolo/oyad174)
Supplement: oyad174_suppl_Supplementary_Materials [file oyad174_suppl_supplementary_materials.zip › SUPPLEMENTAL FIGURE LEGEND.docx]

**SUPPLEMENTAL FIGURE LEGEND**

**Supplemental Figure 1.** Mean baseline Charlson Comorbidity Index scores

Abbreviations: 1L, first line; ICI, immune checkpoint inhibitor.
