# Supplementary material for: Clinical and Economic Outcomes in Patients With Metastatic Urothelial Carcinoma Receiving First-Line Systemic Treatment (the IMPACT UC I Study)
Source: Oncologist. 2023 Jul 11;28(9):790–8. doi: 10.1093/oncolo/oyad174 (PMC10485286; doi:10.1093/oncolo/oyad174)
Supplement: oyad174_suppl_Supplementary_Materials [file oyad174_suppl_supplementary_materials.zip › Supplemental Tables 1 and 2.docx]

**Supplemental Table 1. First-line treatments**

| **Cisplatin-containing therapy** | **Carboplatin-containing therapy** | **ICI monotherapy** | **Nonplatinum-containing therapy (excluding ICIs)** |
| --- | --- | --- | --- |
| - Cisplatin - MVAC - Dose-dense MVAC - Gemcitabine and cisplatin - Paclitaxel (or nab-paclitaxel), gemcitabine, and cisplatin | - Carboplatin - Carboplatin and gemcitabine - Carboplatin, gemcitabine, and paclitaxel (or nab-paclitaxel) | - Atezolizumab - Avelumab - Durvalumab - Nivolumab - Pembrolizumab | - Docetaxel - Doxorubicin - Epirubicin - Erdafitinib - Fluorouracil - Gemcitabine - Gemcitabine and docetaxel - Gemcitabine and paclitaxel (or nab-paclitaxel) - Ifosfamide - Ifosfamide, doxorubicin, and gemcitabine - Methotrexate - Paclitaxel (or nab-paclitaxel) - Vinblastine |

**ICI,** immune checkpoint inhibitor; **MVAC**, methotrexate, vinblastine, doxorubicin, and cisplatin.

**Supplemental Table 2. Baseline comorbidities by 1L treatment cohort**

| **n (%)** | **1L treatment cohort** | | | | |
| --- | --- | --- | --- | --- | --- |
|  | **Cisplatin- containing chemotherapy (n=3247)** | **Carboplatin- containing** **chemotherapy (n=2602)** | **ICI monotherapy**  **(n=1730)** | **Nonplatinum- containing treatment (n=1051)** | **No 1L treatment identified (n=10,258)** |
| **Cerebrovascular disease** | 295 (9.1) | 323 (12.4) | 221 (12.8) | 146 (13.9) | 1500 (14.6) |
| **Chronic obstructive pulmonary disease** | 873 (26.9) | 741 (28.5) | 499 (28.8) | 297 (28.3) | 3249 (31.7) |
| **Congestive heart failure** | 259 (8.0) | 339 (13.0) | 270 (15.6) | 152 (14.5) | 1822 (17.8) |
| **Diabetes (mild to moderate)** | 888 (27.3) | 932 (35.8) | 571 (33.0) | 349 (33.2) | 3439 (33.5) |
| **Diabetes with complications** | 274 (8.4) | 378 (14.5) | 283 (16.4) | 166 (15.8) | 1573 (15.3) |
| **Peripheral vascular disease** | 631 (19.4) | 625 (24.0) | 484 (28.0) | 257 (24.5) | 2751 (26.8) |
| **Renal disease** | 388 (11.9) | 737 (28.3) | 584 (33.8) | 273 (26.0) | 3102 (30.2) |

**1L,** first line; **ICI,** immune checkpoint inhibitor.
